# Risks of Ventricular Arrhythmia and Heart Failure in Carriers of *RBM20* Variants

**DOI:** 10.1161/CIRCGEN.123.004059

**Published:** 2023-08-18

**Authors:** Douglas E. Cannie, Alexandros Protonotarios, Athanasios Bakalakos, Petros Syrris, Massimiliano Lorenzini, Bianca De Stavola, Louise Bjerregaard, Anne M. Dybro, Thomas M. Hey, Frederikke G. Hansen, Marina Navarro Peñalver, Maria G. Crespo-Leiro, Jose M. Larrañaga-Moreira, Fernando de Frutos, Renee Johnson, Thomas A. Slater, Lorenzo Monserrat, Anshuman Sengupta, Luisa Mestroni, Matthew R.G. Taylor, Gianfranco Sinagra, Zofia Bilinska, Itziar Solla-Ruiz, Xabier Arana Achaga, Roberto Barriales-Villa, Pablo Garcia-Pavia, Juan R. Gimeno, Matteo Dal Ferro, Marco Merlo, Karim Wahbi, Diane Fatkin, Jens Mogensen, Torsten B. Rasmussen, Perry M. Elliott

**Affiliations:** Institute of Cardiovascular Science, University College London, United Kingdom (D.E.C., A.P., A.B., P.S., M.L., P.M.E.).; Department of Inherited Cardiovascular Diseases, Barts Heart Centre, St Bartholomew’s Hospital, London, United Kingdom (D.E.C., A.P., A.B., M.L., P.M.E.).; Population, Policy and Practice Research and Teaching Department, UCL Great Ormond Street Institute of Child Health, United Kingdom (B.D.S.).; Department of Cardiology, Aarhus University Hospital, Denmark (L.B., A.M.D., T.B.R.).; Department of Cardiology, Odense University Hospital, Denmark (T.M.H., F.G.H.).; Inherited Cardiac Disease Unit, Hospital Universitario Virgen Arrixaca, Murcia, Spain (M.N.P., J.R.G.).; European Reference Network for Rare and Low Prevalence Complex Diseases of the Heart, Amsterdam, the Netherlands (M.N.P.,F.d.F., P.G.-P., J.R.G., M.D.F., M.M., G.S.).; Unidad de Cardiopatías Familiares e Insuficiencia Cardíaca Avanzada, Complexo Hospitalario Universitario de A Coruña, Instituto de Investigación Biomédica de A Coruña, Servizo Galego de Saúde, Universidade da Coruña, Spain (R.B.-V., M.G.C.-L., J.M.L.-M.).; Heart Failure and Inherited Cardiac Diseases Unit, Department of Cardiology, Hospital Universitario Puerta de Hierro, Instituto Investigación Sanitaria Puerta de Hierro - Segovia de Arana (IDIPHISA), Madrid, Spain (F.d.F., P.G.-P.).; Centro de Investigación Biomédica en Red de Enfermedades Cardiovasculares (CIBERCV) (M.N.P.,F.d.F., R.B.-V., M.G.C.-L., J.M.L.-M., P.G.-P., J.R.G.).; Victor Chang Cardiac Research Institute, Darlinghurst (R.J., D.F.).; School of Clinical Medicine, University of New South Wales (UNSW) Medicine and Health, UNSW Sydney, Kensington, Australia (R.J., D.F.).; Yorkshire Heart Centre, Leeds General Infirmary, United Kingdom (T.A.S., A.S.).; Medical Department, Dilemma Solutions, A Coruña, Spain (L. Monserrat).; Cardiovascular Institute and Adult Medical Genetics Program, University of Colorado Anschutz Medical Campus, Aurora (L. Mestroni, M.R.G.T.).; Cardiothoracovascular Department, Azienda Sanitaria Universitaria Integrata Giuliano Isontina, University of Trieste, Italy (G.S., M.D.F., M.M.).; Unit for Screening Studies in Inherited Cardiovascular Diseases, Cardinal Stefan Wyszynski Institute of Cardiology, Warsaw, Poland (Z.B.).; Department of Cardiology, Hospital Universitario Donostia, Spain (I.S.-R., X.A.A.).; Assistance Publique–Hôpitaux de Paris, Cochin Hospital, Cardiology Department, Université de Paris, Institut Imagine, France (K.W.).; Cardiology Department, St Vincent’s Hospital, Sydney, Australia (D.F.).; Department of Cardiology, Aalborg University Hospital, Aalborg, Denmark (J.M.).

**Keywords:** dilated cardiomyopathy, heart failure, sudden cardiac death

## Abstract

**BACKGROUND:**

Variants in *RBM20* are reported in 2% to 6% of familial cases of dilated cardiomyopathy and may be associated with fatal ventricular arrhythmia and rapid heart failure progression. We sought to determine the risk of adverse events in *RBM20* variant carriers and the impact of sex on outcomes.

**METHODS:**

Consecutive probands and relatives carrying *RBM20* variants were retrospectively recruited from 12 cardiomyopathy units. The primary end point was a composite of malignant ventricular arrhythmia (MVA) and end-stage heart failure (ESHF). MVA and ESHF end points were also analyzed separately and men and women compared. Left ventricular ejection fraction (LVEF) contemporary to MVA was examined. *RBM20* variant carriers with left ventricular systolic dysfunction (*RBM20*_LVSD_) were compared with variant-elusive patients with idiopathic left ventricular systolic dysfunction.

**RESULTS:**

Longitudinal follow-up data were available for 143 *RBM20* variant carriers (71 men; median age, 35.5 years); 7 of 143 had an MVA event at baseline. Thirty of 136 without baseline MVA (22.0%) reached the primary end point, and 16 of 136 (11.8%) had new MVA with no significant difference between men and women (log-rank *P*=0.07 and *P*=0.98, respectively). Twenty of 143 (14.0%) developed ESHF (17 men and 3 women; log-rank *P*<0.001). Four of 10 variant carriers with available LVEF contemporary to MVA had an LVEF >35%. At 5 years, 15 of 67 (22.4%) *RBM20*_LVSD_ versus 7 of 197 (3.6%) patients with idiopathic left ventricular systolic dysfunction had reached the primary end point (log-rank *P*<0.001). *RBM20* variant carriage conferred a 6.0-fold increase in risk of the primary end point.

**CONCLUSIONS:**

*RBM20* variants are associated with a high risk of MVA and ESHF compared with idiopathic left ventricular systolic dysfunction. The risk of MVA in male and female *RBM20* variant carriers is similar, but male sex is strongly associated with ESHF.

Dilated cardiomyopathy (DCM) is defined by left ventricular dilatation and impaired left or biventricular systolic function that is unexplained by abnormal loading conditions or coronary artery disease. It has an estimated population prevalence of 1 in 250 and, in many patients, is a heritable disease.^1^

Significant progress has been made in unraveling the genetic basis of DCM. While common variation and gene-environment interaction contribute to phenotype development,^2,3^ many families with DCM exhibit Mendelian, monogenic inheritance. Over 200 genes have been implicated in DCM pathogenesis, although strong supportive evidence for a causative role is limited to a relatively small number.^4^

Variants in *RBM20* are reported in 2% to 6% of familial cases of DCM.^5^ *RBM20* resides on chromosome 10 and encodes for the RNA-binding motif protein 20, a modifier of post-transcriptional splicing of over 40 genes. It is suggested that *RBM20* variants cause a particularly malignant form of DCM, characterized by fatal ventricular arrhythmia and rapidly progressive left ventricular systolic dysfunction (LVSD).^6,7^ If this is correct, diagnosis of pathogenic *RBM20* variants has major implications for disease management and the counseling of families. In this study, we examine the natural history of *RBM20* variant carriers. Sex differences are well-documented in DCM, including within gene-specific cohorts.^8–10^ We sought to explore these differences in *RBM20* variant carriers. To provide context to our findings, we compare and contrast the characteristics and outcomes of *RBM20* variant carriers with LVSD with patients with idiopathic LVSD (iLVSD) and no identifiable pathogenic gene variant.

## METHODS

Methods are available in the Supplemental Material. Patient-level data will not be made available as consent was not sought for public dissemination and due to concerns that information could be used to identify individuals. The study conforms with the principles of the Declaration of Helsinki,^11^ and participants provided written informed consent for genetic testing. The authors from each center guarantee the integrity of data from their institution and received approval for anonymized patient data collation and analysis.

## RESULTS

**Figure 1.**
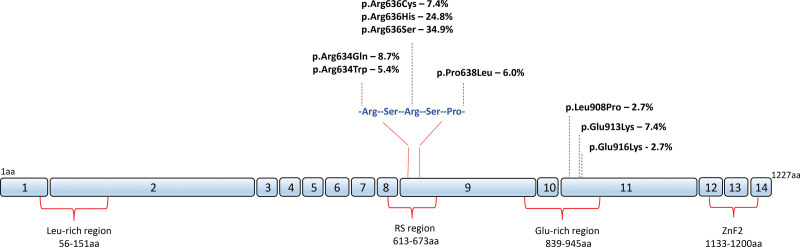
**Schematic representation of *RBM20* and variants comprising the study cohort.** The position and frequency of variants are depicted along the length of the protein. Functional domains containing variants in this study are shown under the protein, and numbers inside blue boxes represent *RBM20* exons. The functionally important arginine-serine-arginine-serine-proline sequence (634-638aa) in the arginine/serine-rich (RS) region is shown in detail. aa indicates amino acid residue; Glu, glutamic acid; Leu, leucine; and ZnF2, zinc finger region 2.

One hundred and fifty-four individuals with likely pathogenic or pathogenic *RBM20* variants were identified. Five were excluded: 1 with hypertrophic cardiomyopathy, 3 with no clinical data, and 1 neonate with a heart failure–related death. The cohort for baseline analysis comprised 149 individuals (32 probands [Table S1] and 117 relatives) from 43 families, 28 of which were represented by ≥2 individuals (Figure S1). All *RBM20* variants were missense variants (130 within the arginine-serine-arginine-serine-proline stretch on exon 9 and 19 in the glutamate-rich region on exon 11; Figure 1; Table S2).

### Baseline Characteristics and Disease Penetrance

Baseline characteristics of *RBM20* variant carriers are summarized in Table 1 and Tables S3 and S4, including columns representing missingness. Seven (4.7%) individuals had malignant ventricular arrhythmia (MVA) before (3/7) or at baseline assessment (4/7). Median (interquartile range [IQR]) age at first evaluation was 35.5 (20.2–47.4) years (41.6 [24.3–49.4] years for women versus 28.0 [17.8–41.5] years for men; *P*=0.001). LVSD was present in 85 (57.0%) patients at or before baseline assessment. Two probands with normal LVEF had unexplained left ventricular dilatation^12^ and nonsustained ventricular tachycardia on Holter monitor. Fifty-five (47.0%) relatives had LVSD at baseline.

**Table 1.**
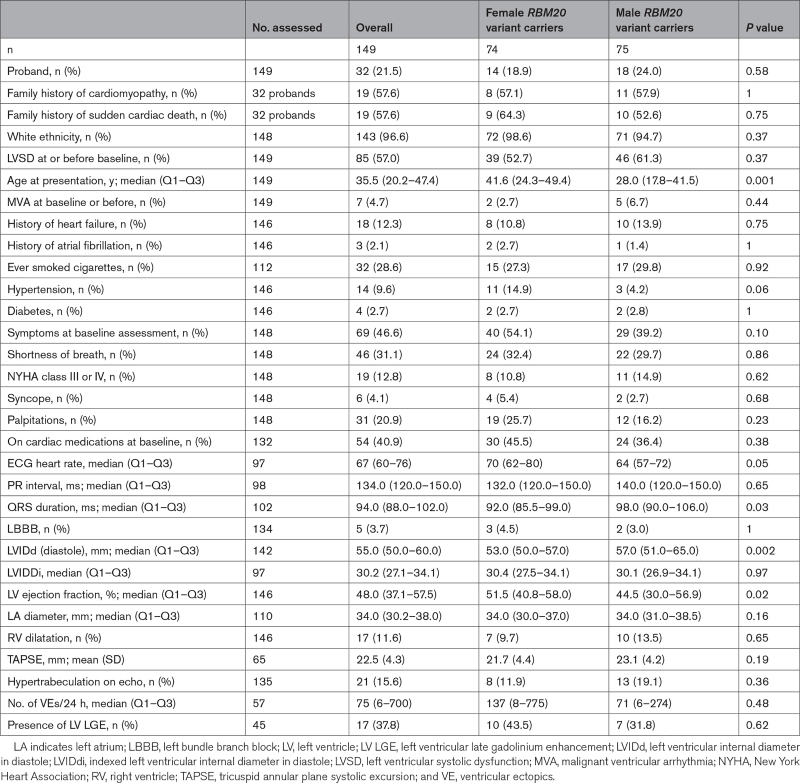
Baseline Characteristics of Male and Female *RBM20* Variant Carriers

Twenty one variant carriers developed new-onset LVSD during follow-up (both probands without LVSD at baseline and 19/61 [31.1%] previously unaffected relatives) with a median (IQR) time to LVSD development of 71 (23–153) months. Estimated median age of disease penetrance in male relatives was 32.1 (27.6–40.1) years and 45.5 (37.9–53.7) years for female relatives (Figure S2).

### Clinical Events

Follow-up data were available for 143 of 149 *RBM20* variant carriers (Table 2). Median (IQR) follow-up was 86 (39–178) months. Thirty-six (25.2%) patients experienced MVA or end-stage heart failure (ESHF) during follow-up. Six of the 7 patients with MVA before or at first evaluation had at least 1 further MVA event during follow-up; 3 of 7 underwent heart transplantation and 1 died from heart failure.

**Table 2.**
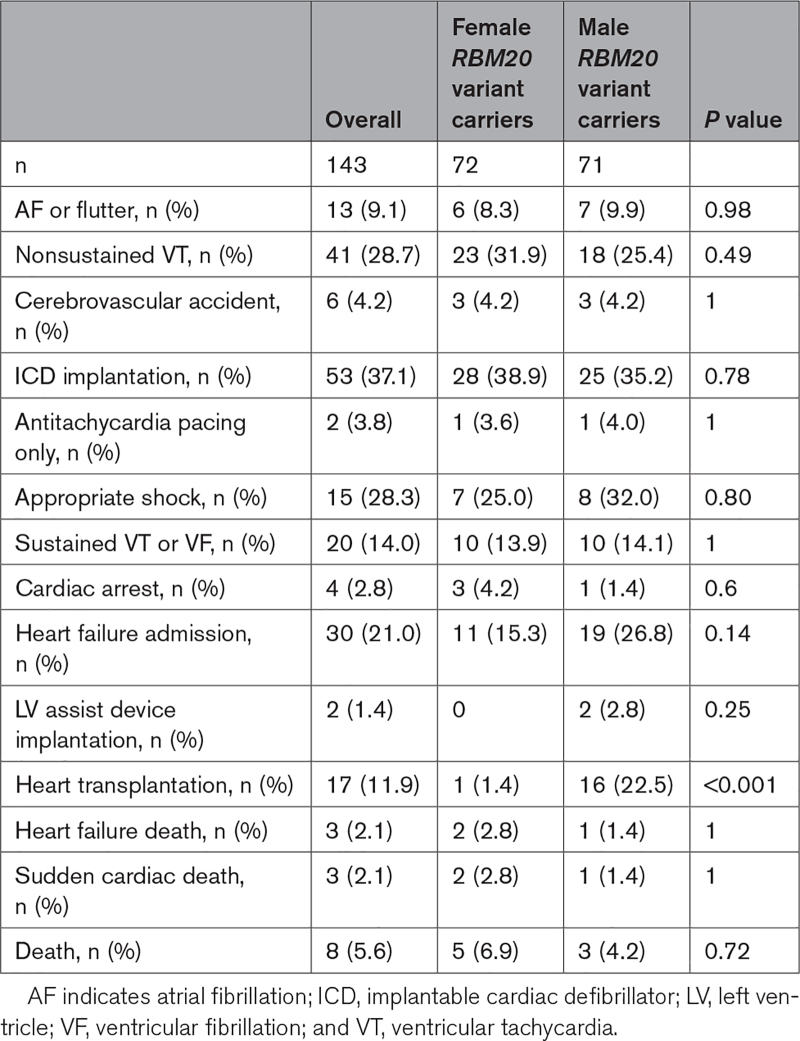
Clinical Outcomes for Male and Female *RBM20* Variant Carriers

Eight *RBM20* variant carriers died during follow-up (3 heart failure–related and 3 sudden cardiac deaths). One woman died of noncardiovascular causes at the age of 86 years, and 1 man died of causes unknown at the age of 48 years.

### Survival Analysis

The 7 individuals with MVA at or before baseline assessment were excluded from analysis of the primary composite and secondary MVA end points but were included in the analysis of the secondary ESHF end point.

**Figure 2.**
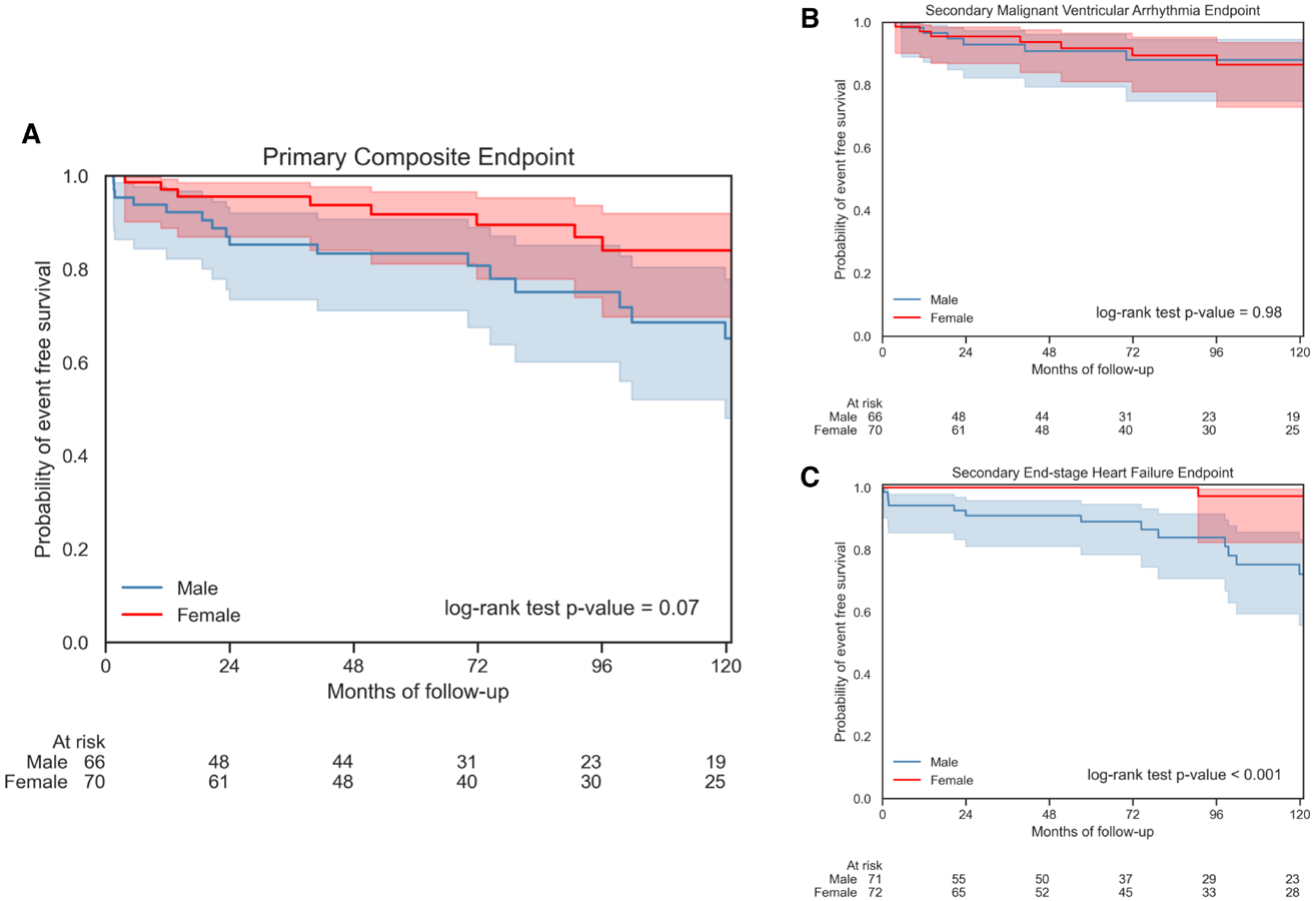
**Survival analyses for *RBM20* variant carriers for the primary composite and secondary end points.** Kaplan-Meier curves comparing outcomes from baseline between men and women for the primary composite end point (**A**), the secondary malignant ventricular arrhythmia end point (**B**), and the secondary end-stage heart failure end point (**C**). For the primary end point, there was a statistically nonsignificant trend toward worse outcomes in men. This trend was driven by men experiencing significantly more end-stage heart failure. Men and women experienced similar rates of malignant ventricular arrhythmia.

Thirty (22.0%) of 136 patients (18 men and 12 women) reached the primary composite end point: 16 had MVA events (7 men and 9 women) and 14 had ESHF events (11 men and 3 women). Only 2 women had adverse events before the age of 40 years (sustained ventricular tachycardia and appropriate implantable cardiac defibrillator [ICD] shock) during follow-up. Differences between men and women for the primary composite and secondary MVA end points were not statistically significant (*P*=0.07 and *P*=0.98, respectively; Figure 2). A Kaplan-Meier analysis of the primary end point from birth, incorporating events before and at baseline assessment (and, therefore, all *RBM20* variant carriers), did show a difference between men and women (*P*<0.001; Figure S3). Competing risk from heart transplantation did not significantly alter the comparison between men and women for the secondary MVA end point (Fine-Gray test, *P*=0.78; Figure S4).

Ten patients who experienced MVA had an LVEF available within 6 months of the event. Median (IQR) contemporary LVEF was 28.5% (23%–38%), and 4 of 10 had a contemporary LVEF >35%. A single patient had a contemporary LVEF >45% (Table S5).

Twenty of 143 patients (14.0%) reached the ESHF end point (17 men and 3 women; *P*<0.001; Figure 2); 17 of 20 had heart transplantations (16 men and 1 woman). One man underwent left ventricular assist device implantation and subsequently had a heart failure–related death. Two female deaths were reported as heart failure related at 62 and 94 years of age. Four men (1 proband) received heart transplants in their teenage years.

### Predictors of Clinical Events in *RBM20* Variant Carriers

Univariable Cox regression identified multiple baseline variables associated with the primary end point (Table S6A) including LVEF (hazard ratio [HR], 1.08 per 1% decrement [95% CI, 1.04–1.12]; *P*<0.001) and left ventricular internal diameter at end diastole (LVIDd; HR, 1.12 per 1 mm increase [95% CI, 1.06–1.18]; *P*<0.001). Variables not included in multivariable analyses due to >10% missing data were history of smoking, cardiac medications at baseline, PR interval, QRS duration, left atrial diameter, tricuspid annular plane systolic excursion, number of ventricular ectopics in 24 hours, and the presence of left ventricular late gadolinium enhancement on cardiac magnetic resonance scan. LVEF and LVIDd demonstrated strong associations with all end points. Multivariable modeling was explored with and without LVIDd given the greater emphasis placed on LVEF in clinical practice and international guidelines.

Multivariable modeling without LVIDd demonstrated that LVEF (HR, 1.08 per 1% decrement [95% CI, 1.04–1.12]; *P*<0.001), carriage of the p.Arg634Gln variant (HR, 5.0 [95% CI, 1.8–13.8]; *P*=0.002), and history of heart failure (HR, 2.6 [95% CI, 1.2–5.7]; *P*=0.02) were independently associated with the primary end point (Table S6B). When LVIDd was included in the model, the independent predictors of the primary end point were LVIDd, history of heart failure, New York Heart Association class III or IV at baseline, and carriage of the p.Arg634Gln variant.

Univariable and multivariable modeling for the secondary MVA and ESHF end points is shown in Tables S7 and S8. As with the primary end point, results differed with and without inclusion of LVIDd. In models excluding LVIDd, LVEF and carriage of the p.Arg634Gln variant were independent predictors of the secondary MVA end point and LVEF was an independent predictor for the secondary ESHF end point in a model with male sex and history of heart failure.

Univariable and multivariable analysis using Fine-Gray subdistribution hazards confirmed these results when accounting for the competing risk of heart transplantation.

### Comparison Between *RBM20*_LVSD_ and iLVSD Cohorts

Seventy-six *RBM20*_LVSD_ patients were compared with 238 iLVSD patients (Tables S9 and S10). Twenty-seven (11.3%) of the iLVSD cohort and 5 (6.6%) of the *RBM20*_LVSD_ cohort had MVA at or before baseline (*P*=0.33). As previously, for analyses of the primary composite and secondary MVA end points but not the ESHF end point, these patients, and those with no follow-up, were removed. Median (IQR) follow-up time for the iLVSD cohort was 40 (25–64) months and for the *RBM20*_LVSD_ cohort was 91 (47–181) months (*P*<0.001). End points were analyzed at 5 years to mitigate for the difference in follow-up time (Table S11)

**Figure 3.**
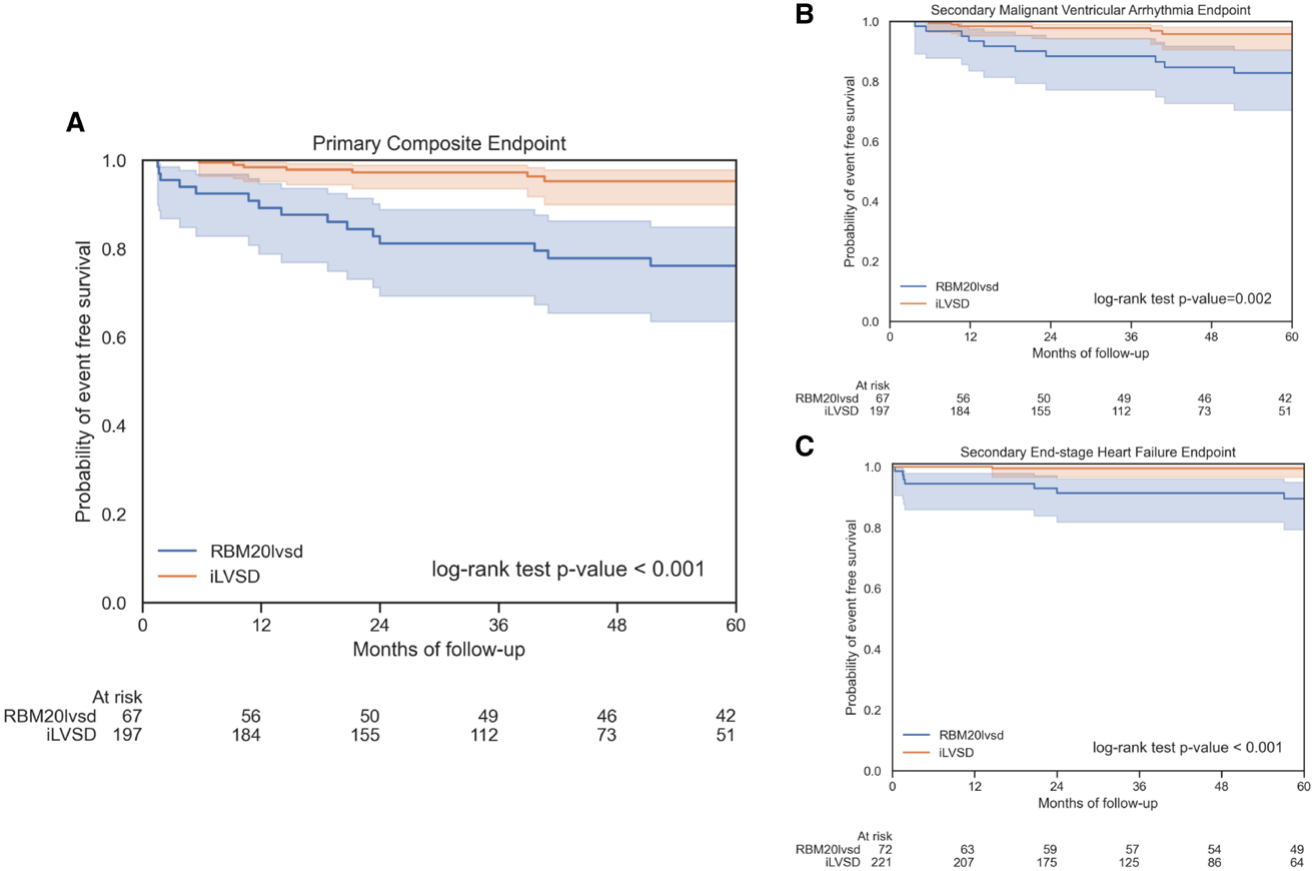
** Survival analyses comparing the *RBM20*_LVSD_ and idiopathic left ventricular systolic dysfunction (iLVSD) cohorts.** Kaplan-Meier curves comparing outcomes from baseline between *RBM20*_LVSD_ and iLVSD cohorts for the primary composite end point (**A**), the secondary malignant ventricular arrhythmia end point (**B**), and the secondary end-stage heart failure end point (**C**). The *RBM20*_LVSD_ cohort had significantly worse outcomes at 5 years for all end points in comparison with the iLVSD cohort.

At 5 years, the primary end point was reached by 15 of 67 (22.4%) patients with *RBM20*_LVSD_ versus 7 of 197 (3.6%) with iLVSD (log-rank *P*<0.001; Figure 3). The incidence of the primary end point in the *RBM20*_LVSD_ cohort was 5.9 per hundred person-years versus 1.1 per hundred person-years for the iLVSD cohort (incidence rate ratio, 5.4). The secondary MVA end point was reached by 10 of 67 (14.9%) patients with *RBM20*_LVSD_ versus 6 of 197 (3.0%) with iLVSD (*P*=0.002), and the secondary ESHF end point reached by 7 of 72 (9.7%) *RBM20*_LVSD_ versus 1 of 221 (0.5%) iLVSD (log-rank *P*<0.001). *RBM20* variant carriage was associated with the primary end point (HR, 6.0 [95% CI, 2.3–15.6]; *P*<0.001) and retained this association when propensity scores were included in a multivariable Cox model (HR, 4.6 [95% CI, 1.6–19.5]; *P*=0.03).

## DISCUSSION

*RBM20* encodes RNA-binding motif protein 20, a trans-acting splicing factor that is highly expressed in striated muscle. It affects the splicing of over 40 genes including *TTN* and other genes important for sarcomeric function, as well as genes involved in cardiomyocyte calcium homeostasis. The interplay between *RBM20* variants and the spliceosome confers intricacy and complexity to the mechanism of *RBM20*-induced DCM.

In this large, multicenter, international study, we show that *RBM20* variants associated with DCM frequently result in adverse events with over 1 in 5 patients reaching the primary composite outcome during a median follow-up of 87 months. Compared with a control group of variant-elusive patients, *RBM20* variants conferred a 6.0-fold increase in the risk of ESHF or MVA in individuals with LVSD at 5 years of follow-up. *RBM20* variants were shown to be highly penetrant in relatives, an important finding when counseling families.

Men with DCM generally have more severe phenotypes and a poorer prognosis than women.^8–10^ In our study, men and women had a similar burden of MVA; however, men experienced earlier and more frequent ESHF events than women, and only 1 woman had a heart transplant. As a result, a trend was seen toward more men reaching the primary composite end point, but this did not reach statistical significance.

Carriage of the p.Arg634Gln variant was seen to confer additional risk, particularly for MVA events. This variant is located within the arginine-serine-arginine-serine-proline stretch where missense mutations result in mislocalization of *RBM20* to the cytoplasm and altered nuclear splicing of the pre-mRNAs of several cardiomyopathy-linked genes. Splicing models suggest that the pathogenicity of *RBM20* missense variants at the same amino acid position could vary even by polarity of the exchanged amino acid.^13^ In the future, variant-level phenotyping may be required to refine risk assessment.

The need for ICD implantation and its timing are common clinical dilemmas in the management of patients with DCM. Cohort studies of variant carriers in genes such as *LMNA* and *FLNC* have shown that the conventional approach of only offering ICD implantation to patients with severe LVSD is inadequate; in these patients, ICD implantation should be considered in patients where LVSD is only mild or even low-normal.^14,15^ Where data were available, nearly half of *RBM20* variant carriers with an MVA event had a contemporary LVEF >35%, but only a single case was seen with a contemporary LVEF >45%. Thus, consideration of ICD implantation in *RBM20* variant carriers with an LVEF ≤45% seems reasonable. Importantly, women should be regarded as carrying the same risk of MVA as men.

Our findings emphasize the importance of family screening. Nearly a third of relatives with no features of cardiomyopathy at baseline developed LVSD during follow-up. Screening should be considered at a young age given that 3 male relatives required heart transplantation in their teens. The identification of *RBM20* variant carriers before phenotype development offers the opportunity for regular risk assessment and timely initiation of medications. Future therapies may mitigate against the development of the malignant *RBM20* phenotype.^5,16^

### Limitations

Participating centers were all specialist cardiomyopathy units leading to potential referral bias. The use of RedCap^17^ aimed to mitigate against unstandardized data collection that can be a flaw in retrospective, multicenter studies. Some study variables were limited by missing data. Ambulatory ECG monitor and cardiac magnetic resonance scan data were particularly prone to missingness, and this prevented their inclusion in multivariable modeling. How variables such as ventricular ectopy count and late gadolinium enhancement burden contribute to risk stratification in *RBM20* variant carriers should be addressed in future studies. Information on ICD programming was not analyzed, and while all centers are expert in device implantation and offer contemporary programming strategies, patients could have been exposed to different practices that may have influenced the frequency of MVA events. Most *RBM20* variant carriers were of white ethnicity, highlighting the importance of further research incorporating patients of other ethnicities.

### Conclusions

*RBM20* variants are highly penetrant for a malignant DCM phenotype and specific *RBM20* variants associate with adverse events. Both sexes are at risk of MVA, and male sex is strongly associated with ESHF. Impairment of LVEF is a major predictor of the risk of MVA. Further research with larger cohorts and functional studies is required to achieve variant-level risk stratification.

## ARTICLE INFORMATION

### Acknowledgments

The authors are grateful to the patients and their families whose contributions have made this work possible. This study was supported by the National Institute for Health Research University College London Hospitals Biomedical Research Centre.

### Sources of Funding

This work has been supported by a British Heart Foundation clinical research training fellowship to Dr Cannie (FS/CRTF/20/24022); a British Heart Foundation clinical research training fellowship to Dr Protonotarios (FS/18/82/34024); grant support from Instituto de Salud Carlos III (CM20/00101) to Dr de Frutos; the National Institutes of Health (NIH; R01HL69071, R01HL116906, R01HL147064, and Clinical and Translational Science Award from the National Center for Advancing Translational Sciences UL1TR001082) and the American Heart Association (17GRNT33670495) to Dr Mestroni; the NIH (1K23HI067915, R01 HL147064, and R01HL109209) to Dr Taylor; DETECTin-HF (Determining the role of clinical and epigenetic risk markers in dilated cardiomyopathies and heart failure) grant from the The European Research Area Network on Cardiovascular Diseases (ERA-CVD) framework, National Centre for Research and Development to Dr Bilinska; and funding from the Victor Chang Cardiac Research Institute and New South Wales Health to Dr Fatkin.

### Disclosures

Dr Sengupta reports speakers fees from Pfizer. Dr Elliott reports consultancies for Pfizer, Sarepta, Bristol Myers Squibb, Biomarin, Leal, and Novo Nordisk. The other authors report no conflict.

### Supplemental Material

Supplemental Methods

Tables S1–S11

Figures S1–S4

References 17–38

## Supplementary Material


