# Supplementary material for: Risks of Ventricular Arrhythmia and Heart Failure in Carriers of RBM20 Variants
Source: Circ Genom Precis Med. 2023 Aug 18;16(5):434–41. doi: 10.1161/CIRCGEN.123.004059 (PMC10581410; doi:10.1161/CIRCGEN.123.004059)
Supplement: Supplementary file 1 [file hcg-16-434-s001.pdf]

Supplemental Material

Supplemental Methods

Study Design and Cohort Composition

This was a multicenter, retrospective longitudinal cohort study. Patients and relatives with *RBM20* variants were recruited from 12 centers in Europe and Australia between 1986 and 2021 (Supplemental Table I). Probands were defined as an index patient with an LVEF < 50% or unexplained left ventricular dilatation. Relatives carrying *RBM20* variants were recruited irrespective of their left ventricular systolic function.

A control cohort of patients with idiopathic LVSD (LVEF < 50% unexplained by coronary artery disease or abnormal loading conditions), consecutively genotyped and returning a negative result (no likely pathogenic or pathogenic (LP/P) variants) between 2015 and 2019 was retrospectively selected from the University College London cardiomyopathy database (iLVSD cohort) for comparison with *RBM20* variant-carriers with LVSD ($RBM20_{LVSD}$ cohort).

Genetic Testing and Variant Classification

For the *RBM20* cohort, genetic testing in probands was undertaken using predominantly next-generation targeted panels at participating centers or accredited diagnostic laboratories. In 2 families (3 *RBM20* variant-carriers), genetic testing was conducted with gene panels in which *FLNC* was not included. In remaining families, genetic testing included all genes in which variants are associated with strong or definitive evidence of association with a DCM phenotype.[4] Variants were classified using American College of Medical Genetics (ACMG)

criteria (Supplemental Table II).[18] Where additional evidence of pathogenicity (e.g. segregation data) was available from the contributing center, variants were reclassified accordingly. Only LP/P *RBM20* variants were included in the analysis. *RBM20* variant-carriers with additional LP/P variants in other genes associated with cardiomyopathy were excluded. Sanger sequencing was used for cascade screening of relatives.

For the iLVSD cohort, next-generation targeted sequencing using panels with a minimum of 81 genes (Supplemental Table IX) was undertaken at an accredited diagnostic laboratory. These panels included all genes in which variants are associated with strong or definitive evidence of association with a DCM phenotype.[4]

Data Collection and Study Variables

Study data were collected independently by each center and managed using REDCap (Research Electronic Data Capture) tools hosted at University College London.[17] Standard data collection procedures, in accordance with general data protection regulation, were followed. Baseline demographics, comorbidities, symptoms, 12-lead electrocardiogram (ECG), transthoracic echocardiogram (TTE), ambulatory Holter ECG recordings and cardiac magnetic resonance (CMR) scan data were collected from clinical records. The baseline phenotypic data comprised the primary dataset used for most analyses.

Study Endpoints

Follow-up time was calculated from date of first evaluation to date of most recent evaluation, heart transplantation or death from any cause. The primary endpoint was a composite of malignant ventricular arrhythmia (MVA) (defined as SCD, aborted SCD, appropriate implantable cardiac defibrillator (ICD) therapy or sustained ventricular tachycardia (VT) or

fibrillation (VF)) and end-stage heart failure (ESHF) (left ventricular assist device implantation (LVAD), heart transplantation or heart failure-related death).[19] MVA and ESHF endpoints were also analysed separately as secondary endpoints. Patients were censored at the time of their first endpoint event during follow-up or at their last evaluation. New onset phenotype development in variant-carriers with normal baseline evaluation was defined as an LVEF < 50% on echocardiogram or CMR scan or occurrence of MVA.

Statistical Analysis

All data were anonymised and statistical analyses performed using SPSS (version 27.0)[20] and the Python programming language (Version 3.8, Python Software Foundation).[21] Continuous variables were tested for normality of distribution by visual inspection of histograms and statistical normality tests (Shapiro-Wilk). Normally distributed variables are expressed as mean ± SD and non-normally distributed variables as median [25th, 75th percentiles]. Categorical variables are reported as counts and percentages, as appropriate.
The *TableOne* library was used for the construction of summary statistics tables and for statistical comparisons.[22] The *Seaborn* and *Matplotlib* libraries were used for data visualization.[23] The *zEpid* library was used to calculate incidence rates.[24]

The *Lifelines* library was used for all time-to-event analyses.[25] Kaplan-Meier plots were used to display the cumulative probability of the occurrence of endpoints, stratified by sex, within the *RBM20* cohort and to compare endpoints between the iLVSD and *RBM20*$_{LVSD}$ cohorts. The log-rank test was used to compare survival. The impact of competing risk from heart transplantation on the incidence of MVA was assessed using Aalen-Johansen and Fine-Gray analyses.[26,27] P-values < 0.05 were considered significant. A cumulative probability plot was used to examine age at disease penetrance.

Univariable Cox regression was used to assess the association of baseline variables with endpoints after observation that the proportional hazards assumption was supported by the data. Where variables had less than 10% of data missing, a stochastic imputation method was used to allow inclusion in multivariable models. The *Sklearn* library (impute.IterativeImputer) was used to perform a total of 10 imputation rounds before returning the imputations computed during the final round.[28] A round was a single imputation of each feature with missing values.

Forward stepwise regression using Cox proportional hazards was used to identify multivariable models associated with adverse events during follow-up and calculate hazard ratios (HR) and 95% confidence intervals (CIs). Indicator variables were created for the three most prevalent *RBM20* variants (p.Arg636Ser, p.Arg636His and p.Arg634Gln) and included in the forward stepwise regression model. The robust sandwich estimator was used to obtain standard errors to deal with correlations within families.

Propensity scores for *RBM20* variant carriage were estimated using plausible modifiers of phenotype (sex, age at presentation, hypertension and diabetes). These scores were included in a multivariable Cox model in order to adjust for differences in baseline characteristics between the $\textit{RBM20}_{\mathrm{LVSD}}$ and iLVSD groups, with bootstrapping estimation (with 1000 replications) of standard errors to allow for uncertainty in the estimated propensity scores.

Supplemental Tables

Centre	Sex	Age at Presentation	Enrolment Year
London	Male	23	1998
London	Female	34	2019
London	Female	23	2019
London	Female	52	2003
London	Male	48	2013
London	Male	58	2004
Murcia	Female	46	2003
Murcia	Male	50	2017
Paris	Female	48	2012
Donostia	Male	42	2018
Warsaw	Male	24	2017
Odense	Female	63	2010
Odense	Male	14	1986
Odense	Male	32	1999
Odense	Male	39	2000
Sydney	Male	74	2005
Trieste	Female	42	1994
Trieste	Female	50	1992
Trieste	Male	49	2019
Madrid	Male	40	2014
Aarhus	Female	37	2002
Aarhus	Male	48	2000
Aarhus	Male	39	2011
Aarhus	Male	37	2000
Aarhus	Female	46	2005
Aarhus	Male	35	2002
Aarhus	Female	41	2020
Aarhus	Male	51	1999
Aarhus	Female	60	2016
Aarhus	Female	50	2015
Aarhus	Male	27	1999
Aarhus	Female	52	2004

Supplemental Table I: Enrolment details of the 32 probands within the *RBM20* cohort.

RBM20 coding DNA sequence location	Protein change	Type of mutation	Location	ClinVar annotation [29]	ACMG classification[18] (including additional data available to contributing centres)	gnomAD[30] MAF	References
c.1906C>A	p.Arg636Ser	Missense	RS region - exon 9	P/LP	LP: PM1, PM2, PM5, PP3, PP5	No entry	6, 31-33
c.1907G>A	p.Arg636His	Missense	RS region - exon 9	P/LP	LP: PM1, PM2, PM5, PP3, PP5	No entry	6,31,34,35
c.1901G>A	p.Arg634Gln	Missense	RS region - exon 9	P	LP: PM1, PM2, PM5, PP3, PP5	1/152378 (0.0006%)	6,31,32
c.2737G>A	p.Glu913Lys	Missense	Glu-rich region - exon 11	P/LP	LP: PS3, PM2, PP1_moderate*, PP3, PP5	No entry	6,7,13,36
c.1906C>T	p.Arg636Cys	Missense	RS region - exon 9	Conflicting evidence	LP: PM1, PM2, PM5, PP3, PP5	No entry	34
C.1913C>T	p.Pro638Leu	Missense	RS region - exon 9	P	LP: PM1, PM2, PM5, PP3, PP5	No entry	6, 13, 31,32,37
c.1900C>T	p.Arg634Trp	Missense	RS region - exon 9	P/LP	LP: PM1, PM2, PM5, PP3, PP5	No entry	34,38
c.2746G>A	p.Glu916Lys	Missense	Glu-rich region - exon 11	LP	LP: PM2, PP3, PP1_moderate*, PP5	No entry	6,7,13,36
c.2723T>C	p.Leu908Pro	Missense	Glu-rich region - exon 11	VUS	LP: PM2, PP3, PP1_moderate*, PP5	No entry	6,7,13,36

Supplemental Table II: *RBM20* variants within the study cohort and their ACMG classification with supporting criteria. Variant nomenclature according to Ensembl transcript ID no: ENST00000369519. *PP1_moderate called for three variants based on available segregation data showing 5 or more informative meioses. Abbreviations: ACMG, American College of Medical Genetics and Genomics; LP, likely pathogenic; MAF, minor allele frequency; P, pathogenic; PM, moderate evidence for pathogenicity; PP, supporting evidence for pathogenicity; PS, strong evidence for pathogenicity; VUS, variant of uncertain significance; ZnF2, zinc finger region 2

	No. assessed	Overall	Female	Male	P-Value
n		32	14	18	
White ethnicity, n (%)	32	31 (96.9)	14 (100.0)	17 (94.4)	1
LVSD at or prior to baseline, n (%)	32	30 (93.8)	13 (92.9)	17 (94.4)	1
Age at presentation, median [Q1,Q3]	32	43.9 [36.3,50.2]	46.7 [41.3,51.2]	39.7 [33.0,48.6]	0.18
MVA at baseline or before, n (%)	32	1 (3.1)	0	1 (5.6)	1
Prior history of heart failure, n (%)	32	12 (37.5)	5 (35.7)	7 (38.9)	1
History of atrial fibrillation, n (%)	32	1 (3.1)	1 (7.1)	0	0.44
Ever smoked cigarettes, n (%)	27	11 (40.7)	6 (50.0)	5 (33.3)	0.45
Hypertension, n (%)	32	4 (12.5)	3 (21.4)	1 (5.6)	0.30
Diabetes Mellitus, n (%)	32	1 (3.1)	1 (7.1)	0	0.44
Symptoms at baseline assessment, n (%)	32	26 (81.2)	10 (71.4)	16 (88.9)	0.37
Shortness of breath, n (%)	32	22 (68.8)	8 (57.1)	14 (77.8)	0.28
NYHA Class III or IV, n (%)	32	11 (34.4)	2 (14.3)	9 (50.0)	0.06
Syncope, n (%)	32	4 (12.5)	2 (14.3)	2 (11.1)	1
Palpitations, n (%)	32	9 (28.1)	5 (35.7)	4 (22.2)	0.45
On cardiac medications at baseline, n (%)	30	19 (63.3)	10 (76.9)	9 (52.9)	0.26
ECG heart rate, median [Q1,Q3]	22	67 [60,88]	65 [62,76]	73 [58,92]	0.53
PR interval (ms), median [Q1,Q3]	20	152.5 [145.8,165.5]	150.0 [127.5,158.2]	160.0 [149.0,169.2]	0.20
QRS duration (ms), median [Q1,Q3]	21	100.0 [90.0,112.0]	95.5 [90.0,99.2]	106.0 [102.5,126.0]	0.02
LBBB, n (%)	28	2 (7.1)	0	2 (13.3)	0.48
LVIDd (mm), median [Q1,Q3]	29	65.0 [59.0,70.0]	59.0 [56.0,64.0]	66.5 [64.5,74.0]	0.002
LVEF (%), median [Q1,Q3]	31	32.5 [23.8,41.0]	37.5 [34.2,44.5]	25.0 [20.0,32.5]	0.003
LA diameter (mm), median [Q1,Q3]	23	39.0 [34.5,42.5]	39.5 [33.8,42.0]	38.0 [35.5,44.5]	0.6
RV dilatation, n (%)	31	7 (22.6)	2 (14.3)	5 (29.4)	0.41
TAPSE (mm), mean (SD)	15	21.2 (5.7)	20.7 (5.5)	22.0 (6.5)	0.70
Hypertrabeculation on echo, n (%)	26	4 (15.4)	1 (8.3)	3 (21.4)	0.60
Number VEs/24 hours , median [Q1,Q3]	16	363 [57,1488]	795 [151,1756]	151 [55,283]	0.28
Presence LV LGE, n (%)	12	8 (66.7)	4 (80.0)	4 (57.1)	0.58

Supplemental Table III: Baseline characteristics of male and female *RBM20* probands. Abbreviations: ECG, electrocardiogram; LA, left atrium; LBBB, left bundle branch block; LVEF, left ventricular ejection fraction; LVIDd, left ventricular internal diameter in diastole; LV LGE, left ventricular late gadolinium enhancement; LVSD, left ventricular systolic dysfunction; MVA, malignant ventricular arrhythmia; NYHA, New York Heart Association; RV, right ventricle; VE, ventricular ectopics.

	No. assessed	Overall	Female	Male	P-Value
n		117	60	57	
White ethnicity, n (%)	116	112 (96.6)	58 (98.3)	54 (94.7)	0.36
LVSD at or prior to baseline, n (%)	117	55 (47.0)	26 (43.3)	29 (50.9)	0.53
Age at presentation, median [Q1,Q3]	117	30.9 [18.9,45.9]	38.2 [21.3,48.4]	23.7 [16.3,36.5]	0.001
MVA at baseline or before, n (%)	117	6 (5.1)	2 (3.3)	4 (7.0)	0.43
Prior history of heart failure, n (%)	114	6 (5.3)	3 (5.0)	3 (5.6)	1
History of atrial fibrillation, n (%)	114	2 (1.8)	1 (1.7)	1 (1.9)	1
Ever smoked cigarettes, n (%)	85	21 (24.7)	9 (20.9)	12 (28.6)	0.57
Hypertension, n (%)	114	10 (8.8)	8 (13.3)	2 (3.7)	0.10
Diabetes Mellitus, n (%)	114	3 (2.6)	1 (1.7)	2 (3.7)	0.60
Symptoms at baseline assessment, n (%)	116	43 (37.1)	30 (50.0)	13 (23.2)	0.005
Shortness of breath, n (%)	116	24 (20.7)	16 (26.7)	8 (14.3)	0.16
NYHA Class III or IV, n (%)	116	8 (6.9)	6 (10.0)	2 (3.6)	0.27
Syncope, n (%)	116	2 (1.7)	2 (3.3)	0	0.50
Palpitations, n (%)	116	22 (19.0)	14 (23.3)	8 (14.3)	0.32
On cardiac medications at baseline, n (%)	102	35 (34.3)	20 (37.7)	15 (30.6)	0.58
ECG heart rate, median [Q1,Q3]	75	67 [59,75]	72 [63,80]	64 [57,69]	0.006
PR interval (ms), median [Q1,Q3]	78	130 [119,144]	129 [120,143]	130 [117,145]	1
QRS duration (ms), median [Q1,Q3]	81	94 [88,100]	92 [85,98]	95 [90,102]	0.14
LBBB, n (%)	106	3 (2.8)	3 (5.6)	0	0.24
LV internal diameter (diastole) (mm), median [Q1,Q3]	113	52.0 [49.0,57.0]	52.0 [48.2,56.0]	55.0 [50.0,61.0]	0.02
LV ejection fraction (%), median [Q1,Q3]	115	52.5 [42.5,58.5]	55.0 [45.0,60.0]	50.0 [40.0,58.0]	0.10
LA diameter (mm), median [Q1,Q3]	87	34.0 [29.5,37.0]	33.0 [28.5,35.5]	34.0 [30.8,38.0]	0.15
RV dilatation, n (%)	115	10 (8.7)	5 (8.6)	5 (8.8)	1
TAPSE (mm), mean (SD)	50	22.8 (3.8)	22.1 (3.9)	23.3 (3.7)	0.28
Hypertrabeculation on echo, n (%)	109	17 (15.6)	7 (12.7)	10 (18.5)	0.57
Number VEs/24 hours, median [Q1,Q3]	41	27 [4,435]	41 [3,589]	15.0 [5,201]	0.84
Presence LV LGE, n (%)	33	9 (27.3)	6 (33.3)	3 (20.0)	0.46

Supplemental Table IV: Baseline characteristics of male and female *RBM20* relatives. Abbreviations: ECG, electrocardiogram; LA, left atrium; LBBB, left bundle branch block; LVEF, left ventricular ejection fraction; LVIDd, left ventricular internal diameter in diastole; LV LGE, left ventricular late gadolinium enhancement; LVSD, left ventricular systolic dysfunction; MVA, malignant ventricular arrhythmia; NYHA, New York Heart Association; RV, right ventricle; VE, ventricular ectopics.

Proband status	Age at first encounter (years)	Sex	Baseline LVEF (%)	Age at MVA (years)	LVEF contemporary to MVA (%)	Type of MVA event
Relative	44	Female	65	44	65	Sustained VT or VF
Relative	55	Male	40	55	40	Appropriate ICD therapy
Proband	24	Male	25	28	40	Appropriate ICD therapy
Relative	17	Female	43	29	37	Appropriate ICD therapy
Proband	39	Male	30	39	30	Appropriate ICD therapy
Proband	41	Female	32	42	30	Appropriate ICD therapy
Proband	23	Female	27	23	25	Appropriate ICD therapy
Relative	55	Male	23	55	23	Appropriate ICD therapy
Relative	44	Male	16	44	16	Sustained VT or VF
Proband	39	Male	13	39	13	Sustained VT or VF

Supplemental Table V: *RBM20* variant carriers with LVEF available contemporary to MVA event. Table showing the details of ten *RBM20* variant carriers with LVEF available within 6 months of an MVA event. Four patients had a contemporary LVEF > 35%. LVEF, left ventricular ejection fraction; MVA, malignant ventricular arrhythmia; ICD, implantable cardiac defibrillator; VT, ventricular tachycardia.

Variable	Univariable HR [95% CI]	Univariable p value
Baseline LVIDd (per 1mm increase on TTE)	1.12 [1.06 – 1.18]	< 0.001
Baseline LVEF (per 1% decrement on TTE)	1.08 [1.04 – 1.12]	< 0.001
NYHA class 3-4 at baseline	6.4 [2.8 – 15.1]	< 0.001
Baseline LAD (per 1mm increase on TTE)	1.15 [1.09 – 1.21]	< 0.001
Prior history of heart failure	5.2 [2.4 – 11.1]	< 0.001
Proband status	4.5 [1.9 – 10.9]	< 0.001
QRS duration (per 1mm increase on ECG)	1.05 [1.02 – 1.08]	< 0.001
PR interval (per 1mm increase on ECG)	1.03 [1.01 – 1.04]	< 0.001
Cardiac symptoms at baseline assessment	4.1 [1.6 – 10.3]	0.003
Presence of ventricular ectopy on baseline ECG	3.2 [1.4 – 7.2]	0.005
NSVT detected during follow-up	2.3 [1.1 – 4.5]	0.02
Carriage of p.Arg634Gln	1.8 [0.6 – 5.6]	0.32

Variable	Multivariable HR [95% CI]	Multivariable p value	Multivariable HR [95% CI], LVIDd included	Multivariable p value, LVIDd included
Baseline LVIDd (per 1mm increase on TTE)			1.10 [1.05 – 1.15]	< 0.001
Baseline LVEF (per 1% decrement on TTE)	1.08 [1.04 – 1.12]	< 0.001		
NYHA Class 3-4 at baseline			3.6 [1.7 – 7.7]	<0.001
Prior history of heart failure	2.6 [1.2 – 5.7]	0.02	2.8 [1.2 – 6.7]	0.02
Carriage of p.Arg634Gln	5.0 [1.8 – 13.8]	0.002	3.2 [1.5 – 7.0]	0.003

Supplemental Tables VIa and VIb: Univariable and multivariable predictors of the primary composite endpoint. Carriage of the p.Arg634Gln variant was not associated with the primary endpoint on univariable analysis but was selected by forward stepwise regression as part of the multivariable models. Abbreviations: ECG, electrocardiogram; HR, hazard ratio; LAD, left atrial diameter; LVEF, left ventricular ejection fraction; LVIDd, left ventricular internal diameter at end diastole; NYHA, New York Heart failure Association; NSVT, non-sustained ventricular tachycardia; TTE, transthoracic echocardiogram.

Variable	Univariable HR [95% CI]	Univariable p value
Baseline LVIDd (per 1mm increase on TTE)	1.12 [1.06 – 1.18]	< 0.001
Baseline LVEF (per 1% decrement on TTE)	1.08 [1.05 – 1.12]	< 0.001
Baseline LAD (per 1mm increase on TTE)	1.13 [1.07 – 1.19]	< 0.001
Prior history of heart failure	5.5 [2.0 – 15.6]	0.001
PR interval (per 1mm increase on ECG)	1.02 [1.01 – 1.04]	0.004
NYHA class 3-4 at baseline	4.8 [1.6 – 13.9]	0.004
Proband status	5.2 [1.6 – 16.7]	0.006
Presence of ventricular ectopy on baseline ECG	4.5 [1.3 – 16.0]	0.02
Cardiac symptoms at baseline assessment	3.7 [1.2 – 11.4]	0.02
QRS duration (per 1mm increase on ECG)	1.03 [1.00 – 1.06]	0.03
NSVT detected during follow-up	3.0 [1.1 – 8.2]	0.03
Presence LGE (first available CMR scan)	9.9 [1.3 – 76.6]	0.03
Carriage of p.Arg634Gln	3.54 [1.03 – 12.2]	0.04
Male sex	0.99 [0.36 – 2.72]	0.98

Variable	Multivariable HR [95% CI]	Multi-variable p value	Multivariable HR [95% CI], LVIDd included	Multivariable p value, LVIDd included
Baseline LVIDd (per 1mm increase on TTE)			1.17 [1.09 – 1.25]	<0.001
Baseline LVEF (per 1% decrement on TTE)	1.09 [1.06 – 1.13]	<0.001		
Male sex			0.22 [0.05 – 1.02]	0.05
Carriage of p.Arg634Gln	11.7 [4.0 – 34.1]	<0.001		

Supplemental Tables VIIa and VIIb: Univariable and multivariable predictors of the secondary malignant ventricular arrhythmia endpoint. Abbreviations: CMR, cardiac magnetic resonance; ECG, electrocardiogram; HR, hazard ratio; LAD, left atrial diameter; LGE, late gadolinium enhancement; LVEF, left ventricular ejection fraction; LVIDd, left ventricular internal diameter at end diastole; NYHA, New York Heart failure Association; NSVT, non-sustained ventricular tachycardia; TTE, transthoracic echocardiogram.

Variable	Univariable HR [95% CI]	Univariable p value
Baseline LVIDd (per 1mm increase on TTE)	1.09 [1.05 – 1.14]	< 0.001
QTc, Bazzett (per 1ms increase on baseline ECG)	1.03 [1.02 – 1.05]	< 0.001
Baseline LVEF (per 1% decrement on TTE)	1.08 [1.03 – 1.12]	< 0.001
QRS duration (per 1mm increase on ECG)	1.04 [1.02 – 1.06]	< 0.001
Presence LBBB on baseline ECG	4.5 [2.4 – 11.2]	0.001
Baseline LAD (per 1mm increase on TTE)	1.14 [1.05 – 1.23]	0.001
NYHA class 3-4 at baseline	5.2 [1.7 – 15.6]	0.003
Male sex	7.2 [1.7 – 30.2]	0.007
Prior history of heart failure	5.0 [1.5 – 17.3]	0.01
PR interval (per 1mm increase on ECG)	1.04 [1.01 – 1.07]	0.01
Proband status	3.0 [1.2 – 7.3]	0.02
Cardiac symptoms at baseline assessment	4.2 [1.2 – 15.5]	0.03

Variable	Multivariable HR [95% CI]	Multi-variable p value	Multi-variable HR [95% CI], LVIDd included	Multivariable p value, LVIDd included
Baseline LVIDd (per 1mm increase on TTE)			1.10 [1.05 – 1.14]	< 0.001
Baseline LVEF (per 1% decrement on TTE)	1.05 [1.01 – 1.10]	0.02		
Male sex	4.8 [0.96 – 23.7]	0.06		
Prior history of heart failure	2.9 [0.9 – 9.5]	0.08		

Supplemental Tables VIIIa and VIIIb: Univariable and multivariable predictors of the secondary end-stage heart failure endpoint. It was not possible to create a multivariable model when LVIDd was included in forward stepwise regression analysis. Abbreviations: ECG, electrocardiogram; HR, hazard ratio; LAD, left atrial diameter; LVEF, left ventricular ejection fraction; LVIDd, left ventricular internal diameter at end diastole; NYHA, New York Heart failure Association; TTE, transthoracic echocardiogram.

ABCC9, ACTA1, ACTC1, ACTN2, ALMS1, ANKRD1, BAG3, BRAF, CAV3, CRYAB, CSRP3, CTF1, DES, DMD, DNAJC19, DOLK, DSC2, DSG2, DSP, EMD, EYA4, FHL2, FHOD3, FKRP, FKTN, FLNC, FOXD4, GAA, GATA4, GATA6,GATAD1, GLA, GLB1, HFE, JUP, KCNJ2, KCNJ8, LAMA2, LAMA4, LAMP2, LDB3, LMNA, MURC, MYBPC3, MYH6, MYH7, MYL2,MYL3, MYOT, MYPN, NEBL, NEXN, NKX2-5, PDLIM3, PKP2, PLN, PRDM16, PSEN1, PSEN2, PTPN11, RAF1, RBM20, RYR2,SCN5A, SGCA, SGCB, SGCD, SLC22A5, TAZ, TBX20, TCAP, TMEM43, TMPO, TNNC1, TNNI3, TNNT2, TPM1, TTN, TTR, TXNRD2,VCL.

Supplemental Table IX: Genes included in an 81-gene dilated cardiomyopathy panel. All idiopathic left ventricular systolic dysfunction patients (iLVSD comparator cohort) underwent the 81-gene panel as a minimum.

	No. assessed	Overall	iLVSD	$RBM20_{LVSD}$	P-Value
n		314	238	76	
Sex, n (%)	314	195 (62.1)	152 (63.9)	43 (56.6)	0.32
Proband, n (%)	314	253 (80.6)	224 (94.1)	29 (38.2)	<0.001
Family history of cardiomyopathy, n (%)	253 probands	38 (15.0)	20 (8.9)	18 (62.1)	< 0.001
Family history of sudden cardiac death, n (%)	253 probands	57 (22.5)	40 (17.9)	17 (58.6)	<0.001
White ethnicity, n (%)	236	182 (77.1)	109 (68.1)	73 (96.1)	<0.001
Age at presentation, median [Q1,Q3]	314	50.7 [37.3,60.0]	53.2 [42.1,62.2]	38.9 [23.7,49.8]	<0.001
MVA at baseline or before, n (%)	314	32 (10.2)	27 (11.3)	5 (6.6)	0.33
Prior history of heart failure, n (%)	314	91 (29.0)	73 (30.7)	18 (23.7)	0.31
History of atrial fibrillation, n (%)	314	33 (10.5)	30 (12.6)	3 (3.9)	0.05
Ever smoked cigarettes, n (%)	260	105 (40.4)	85 (41.9)	20 (35.1)	0.44
Hypertension, n (%)	314	80 (25.5)	73 (30.7)	7 (9.2)	<0.001
Diabetes Mellitus, n (%)	314	41 (13.1)	39 (16.4)	2 (2.6)	0.004
Symptoms at baseline assessment, n (%)	314	206 (65.6)	159 (66.8)	47 (61.8)	0.51
NYHA Class III or IV, n (%)	314	51 (16.2)	32 (13.4)	19 (25.0)	0.03
On cardiac medications at baseline, n (%)	310	202 (65.2)	155 (65.1)	47 (65.3)	1
ECG heart rate, median [Q1,Q3]	244	70 [60,84]	70 [60,83]	70 [60,88]	0.71
PR interval (ms), median [Q1,Q3]	203	166.0 [148.0,183.0]	171.0 [153.0,188.2]	144.0 [124.0,160.0]	<0.001
QRS duration (ms), median [Q1,Q3]	231	109.0 [98.0,143.5]	114.0 [101.0,154.0]	99.0 [90.0,105.8]	<0.001
LBBB, n (%)	304	75 (24.7)	71 (30.0)	4 (6.0)	<0.001
LVIDd (mm), median [Q1,Q3]	301	60.0 [55.0,66.0]	60.0 [54.0,66.0]	60.0 [56.0,66.0]	0.19
LVEF (%), median [Q1,Q3]	314	35.0 [27.5,42.9]	35.0 [27.5,42.9]	37.5 [26.5,42.6]	0.57
LA diameter (mm), median [Q1,Q3]	240	40.0 [36.0,47.0]	41.5 [37.0,47.0]	37.5 [34.0,42.2]	0.002
RV dilatation, n (%)	314	83 (26.4)	67 (28.2)	16 (21.1)	0.28
TAPSE (mm), median [Q1,Q3]	201	20.0 [16.0,23.0]	19.1 [15.0,22.2]	21.0 [18.0,25.0]	0.02
Hypertrabeculation on echo, n (%)	291	27 (9.3)	19 (8.4)	8 (12.1)	0.51
Number VEs/24 hours, median [Q1,Q3]	164	255 [14,1925]	341 [15,2318]	129 [13,833]	0.54
Presence LV LGE, n (%)	224	130 (58.0)	116 (58.0)	14 (58.3)	1

Supplemental Table X: Baseline characteristics of $\mathit{RBM20}_{LVSD}$ and iLVSD cohorts. Abbreviations: ECG, electrocardiogram; LA, left atrium; LBBB, left bundle branch block; LVEF, left ventricular ejection fraction; LVIDd, left ventricular internal diameter in diastole; LV LGE, left ventricular late gadolinium enhancement; LVSD, left ventricular systolic dysfunction; MVA, malignant ventricular arrhythmia; NYHA, New York Heart Association; RV, right ventricle; VE, ventricular ectopics.

	Overall	iLVSD	*RBM20*$_{LVSD}$	P-Value
n	264	197	67	
AF or Flutter, n (%)	46 (17.4)	41 (20.8)	5 (7.5)	0.02
Non-sustained VT, n (%)	73 (27.7)	48 (24.4)	25 (37.3)	0.0
Cerebrovascular Accident, n (%)	6 (2.3)	3 (1.5)	3 (4.5)	0.173
ICD implantation, n (%)	78 (29.5)	53 (26.9)	25 (37.3)	0.145
Anti-tachycardia pacing only, n (%)	1 (1.0)	0	1 (2.6)	0.392
Appropriate shock, n (%)	6 (6.2)	0	6 (15.8)	0.003
Sustained VT or VF, n (%)	15 (5.7)	6 (3.0)	9 (13.4)	0.004
Cardiac arrest, n (%)	6 (2.3)	4 (2.0)	2 (3.0)	0.646
LV Assist Device Implantation, n (%)	1 (0.4)	1 (0.5)	0	1
Heart Transplantation, n (%)	5 (1.9)	0	5 (7.5)	0.001
Heart Failure Death, n (%)	0	0	0	N/A
Sudden Cardiac Death, n (%)	3 (1.1)	2 (1.0)	1 (1.5)	1
Death, n (%)	10 (3.8)	8 (4.1)	2 (3.0)	1

Supplemental Table XI: Outcomes at 5 years of follow-up for *RBM20*$_{LVSD}$ and iLVSD cohorts. Abbreviations: AF, atrial fibrillation; ICD, implantable cardiac defibrillator; iLVSD, idiopathic left ventricular systolic dysfunction; *RBM20*$_{LVSD}$, *RBM20* variant carriers with left ventricular systolic dysfunction; VF, ventricular fibrillation; VT, ventricular tachycardia.

Supplemental Figures

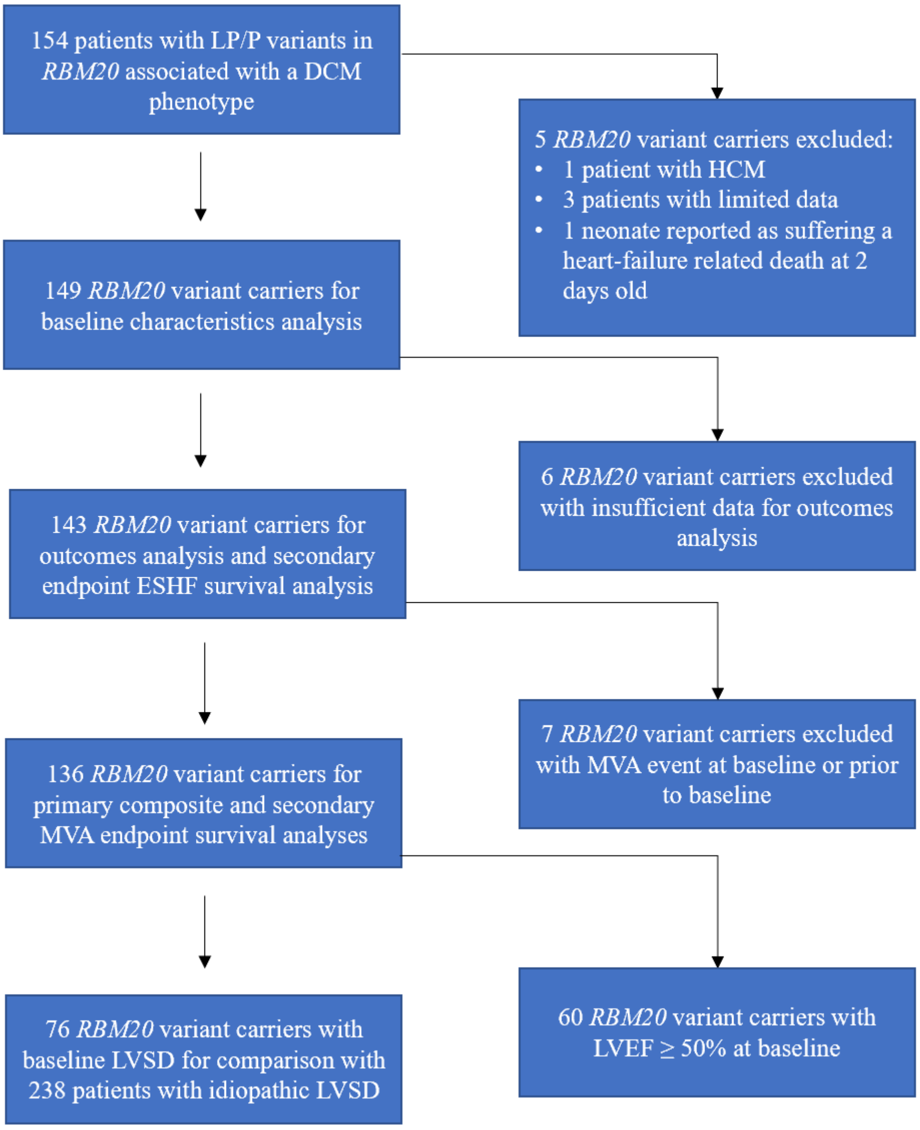

Supplemental Figure I: Flow chart depicting the analysis of *RBM20* variant-carriers.
A detailed overview of the composition of the *RBM20* variant-carrier cohort at the different stages of analysis. Abbreviations: DCM, dilated cardiomyopathy; ESHF, end-stage heart failure; HCM, hypertrophic cardiomyopathy; LP/P, likely pathogenic or pathogenic; LVEF, left ventricular ejection fraction; LVSD; left ventricular systolic dysfunction (LVEF < 50%) MVA, malignant ventricular arrhythmia.

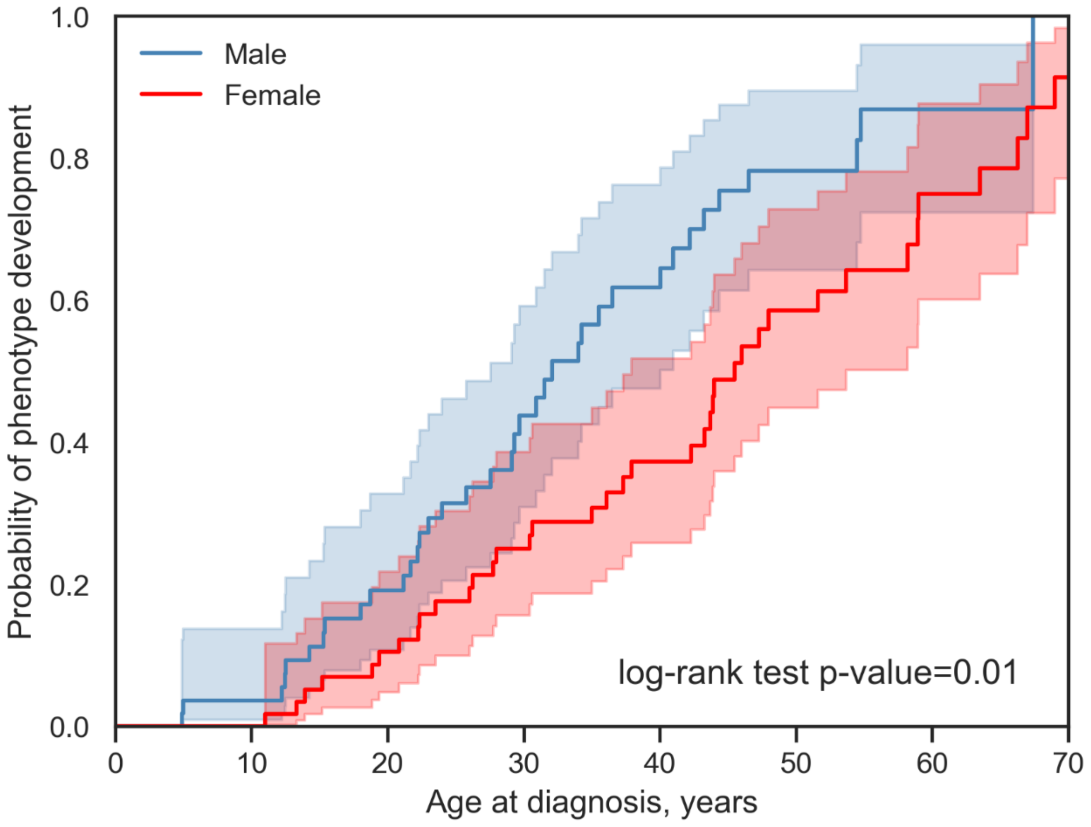

At risk								
Male	55	52	40	22	14	5	1	0
Female	58	58	51	40	28	15	7	2

Supplemental Figure II: Disease penetrance in non-proband *RBM20* variant-carriers. Cumulative probability plots for male and female relatives depicting age (years) at which *RBM20* variant-carriage manifested as a clinical phenotype (left ventricular systolic dysfunction or malignant ventricular arrhythmia). Median age of disease penetrance for males was 32.1 [27.6 – 40.1] years versus 45.5 [37.9 – 53.7] years for females.

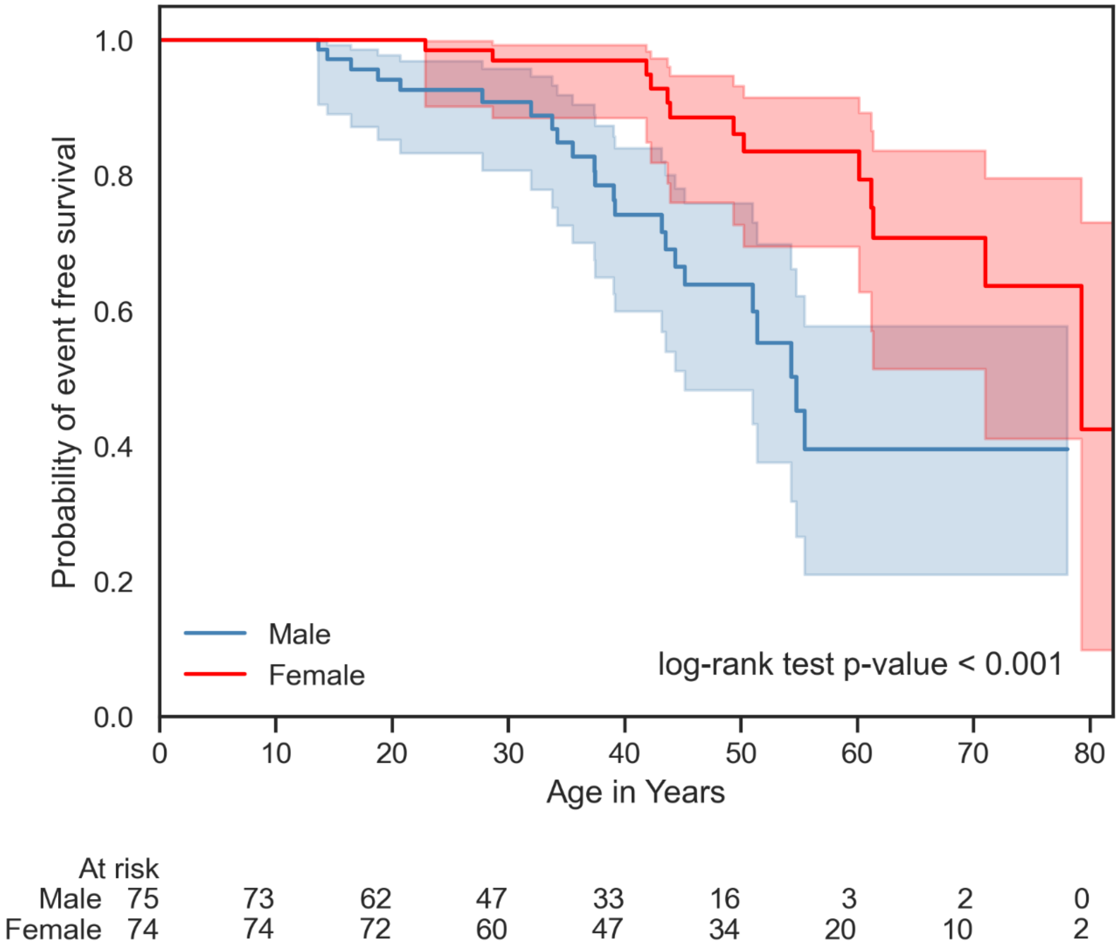

Supplemental Figure III: Survival Analysis from birth for *RBM20* variant-carriers
Kaplan Meier curves illustrating outcomes for male and female *RBM20* variant-carriers for the primary composite endpoint from birth. Data includes events that occurred prior to follow-up or at baseline. Males suffer the primary outcome earlier and more frequently than females.

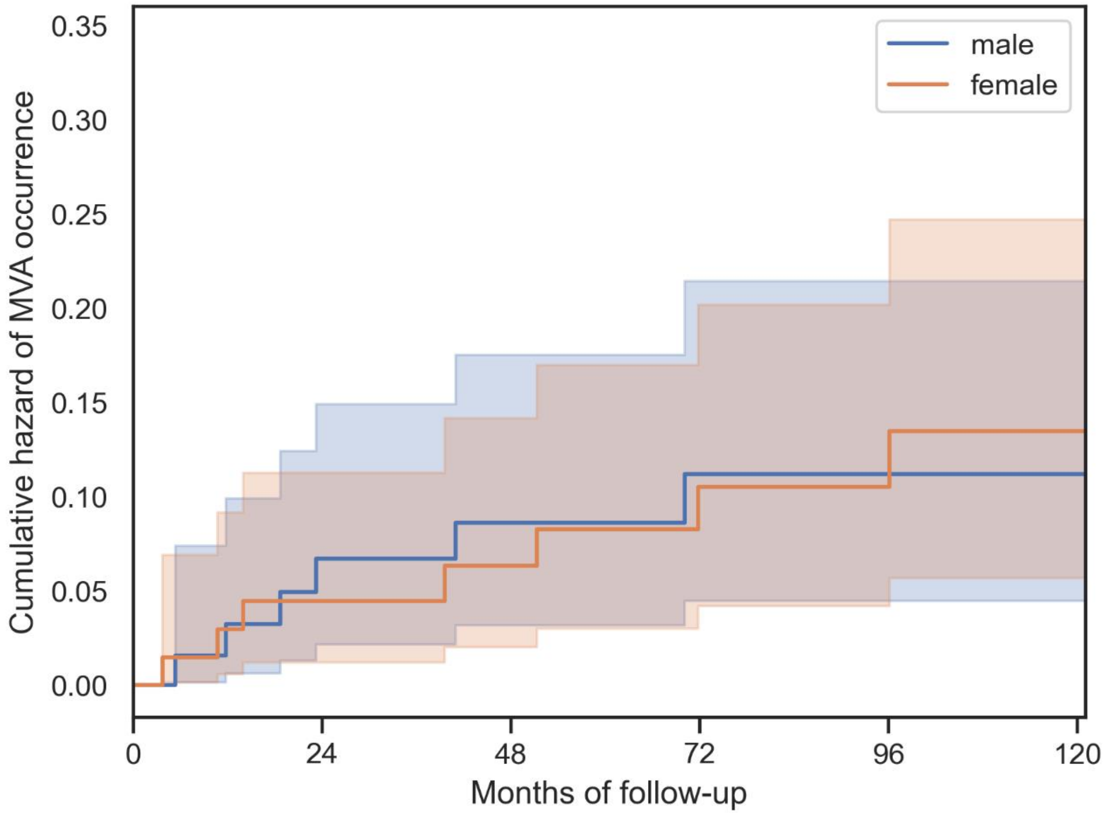

At risk						
male	66	48	44	31	23	19
female	70	61	48	40	30	25

Supplemental Figure IV: Competing risk in the assessment of malignant ventricular arrhythmia. Aalen-Johansen analysis was conducted to assess the influence of competing risk from the disproportionately high number of male versus female heart transplants on the secondary malignant ventricular arrhythmia outcome. Aalen-Johansen curves for males and females appear similar, with a Fine-Gray test p-value of 0.78, suggesting minimal impact of competing risk from male heart transplants on the observed results.
